# Eye Care Practitioners Are Key Influencer for the Use of Myopia Control Intervention

**DOI:** 10.3389/fpubh.2022.854654

**Published:** 2022-03-29

**Authors:** Adeline Yang, Bao Y. Pang, Pushpaja Vasudevan, Björn Drobe

**Affiliations:** ^1^Essilor R&D, Essilor International, Singapore, Singapore; ^2^School of Health Sciences, Ngee Ann Polytechnic, Singapore, Singapore

**Keywords:** myopia, myopia management, parental awareness, questionnaire, eye care practice, opinion of parents, opinion of eye care practitioners

## Abstract

**Background:**

The study sought to investigate the self-reported practices of Singaporean eye care practitioners on myopia management and the interaction between eye care practitioners and parents.

**Methods:**

Self-reported questionnaire (1) to eye care practitioners to understand their clinical practice behavior, their opinion in myopia management (2) to parents on their knowledge of myopia control products and interaction with eye care practitioners.

**Results:**

80.0% of eye care practitioners prescribe myopia control in their practice but only 33.1% of eye care practitioners prescribed myopia control interventions during the first visit, and only 41.4% of parents were recommended myopia control interventions by eye care practitioners, of which 75.6% followed the recommendations of eye care practitioners. Eye care practitioners (53.1%) prefer atropine the most and parents prefer controlling the amount of time doing near work (54.5%) and outdoor activities (52.5%). Eye care practitioners had the highest influence on the choice of vision correction with 78.8% of parents choosing to follow them. 66.9% of eye care practitioners did not prescribe myopia control interventions during the first visit as they lack myopia progression data from the patient. Eye care practitioners felt that more education on myopia control products (57.7%), hands-on workshops (47.7%) and management of children (44.6%) would encourage them to use myopia control interventions more frequently. 40.0% of the eye care practitioners were concerned about the cost of myopia control products.

**Conclusions:**

Eye care practitioners strongly influence parents to uptake myopia control interventions. More education and hands-on workshops on myopia and children management can help encourage the use of myopia control interventions by eye care practitioners.

## Introduction

Myopia prevalence is on the rise, and its trajectory is not slowing down worldwide (1, 2). Myopia is a global public health issue, and with the increase of myopia prevalence, the risk of sight-related pathologies and impairment will increase as well (3–5). Myopia has an impact on public healthcare and the economy (6, 7). Several studies showed that myopia control interventions effectively slowed down the progression of myopia, reducing the severity of myopia endpoint (8–13). Guidelines were even developed to help several of these interventions to be implemented within eye-care practice (14, 15).

Despite available evidence showing the efficacy of interventions for myopia control, the adoption of these interventions by parents and eye care practitioners has been slow. A global survey found that in 2015, 68% of eye care practitioners still prescribed single vision spectacle or contact lenses as the primary mode of correction for myopic patients (16). The main reason for not prescribing myopia control interventions was the high cost of these products, inadequate information on these products, and unpredictability of outcomes (16). A later study in 2019 showed that 52% still prescribe single vision lenses, an improvement from 2015 (17). Another study in Australia found that the absence of regulatory approval poses a concern about medico-legal aspects of prescribing interventions other than conventional glasses, with 50% of the respondents prescribing normal spectacles (single vision lenses) (18).

Singapore is one of the most myopic nations globally, with a myopia prevalence of 81.6% and high myopia prevalence of 13.1% in young adults (19). Though there were studies conducted globally, it is interesting to examine the trends of myopia management amongst eye care practitioners and their interaction with parents in Singapore. This study sought to investigate the self-reported practices of eye care practitioners on myopia management and the interaction between eye care practitioners and parents in Singapore.

## Materials and Methods

### Questionnaire Design for Eye Care Practitioners

The questionnaire was developed to assess:

1. the self-reported clinical practice behavior and opinion of eye care practitioners in myopia management.

2. the perception of eye care practitioners in promoting myopia control interventions to understand the barriers.

A self-administered, internet-based cross-sectional survey in English was distributed using SurveyMonkey (Palo Alto, California, USA) through various professional bodies in Singapore to reach eye care practitioners (optometrist, dispensing opticians, ophthalmologists). The questionnaire comprised 10 questions relating to the self-reported clinical management behaviors of practitioners for myopia.

- What is your profession? (Optician, optometrist, ophthalmologist, student in the eye care course)
- Are you an optical shop/ clinic owner? (Yes, no)
- Do you prescribe Myopia Control interventions? (Yes, No)
- If you do prescribe, may I know what do you prescribe to your customers/ patients? (Multiple options could be selected)
  ° Atropine
  ° Myopia control spectacle lenses
  ° Orthokeratology lenses
  ° Multifocal soft contact lenses
  ° Contact lenses [Soft/RGP]
- Do you prescribe Myopia Control the moment the child has myopia on the first visit? (yes, no)
- May I know the reason for prescribing or not on the first visit? (free text)
- What is preventing you from using Myopia Control on the first visit? (multiple options could be selected)
  ° limited by parent's budget
  ° lack of confidence/experience to prescribe
  ° too much chair time/ too much time spent explaining Myopia control
  ° not knowing enough about myopia control [lack of information]
  ° lack of trust from parents
  ° lack of products to recommend
  ° lack of support from the lens company
  ° the cost price is too high
  ° lack of education to the parents
  ° lack of confidence to manage children
  ° due to unpredictable outcomes
  ° safety of product
  ° limited access to instrumentation [e.g., To prescribe orthokeratology lenses, a corneal topographer is needed]
- What would be your most preferred option to prescribe to your patient when it comes to Myopia Control? (Ranking: Atropine, Myopia control spectacles lenses, multifocal soft contact lenses, orthokeratology lens)
- What would encourage you to fit Myopia Control Interventions more often? (multiple options could be selected)
  ° Education and confidence [product update, myopia management]
  ° experience [having workshops to practice more often]
  ° having safer products
  ° more product choice
  ° cheaper products, education to manage children
  ° guideline from government
  ° Please specify other reasons if not stated above (free text).

### Questionnaire Design for Parents

Another questionnaire was designed to assess:

1. the knowledge of parents about myopia control products.

2. the interaction between parents and eye care practitioners.

The questionnaire was self-administered, internet-based cross-sectional survey in English was distributed using Google Forms (Google Inc., California, USA) through parents' networks in schools and social media to reach Singaporean parents with myopic children. The survey for parents comprises seven questions related to their opinion about myopia management and experience with practitioners.

- Do you have a child/children with myopia (shortsightedness)? (Yes, No)
- What is your child/children using to correct their vision? (Normal spectacles, orthokeratology lenses, myopia control spectacle lenses, atropine, multifocal soft contact lenses, normal soft contact lenses, RGP [hard lenses], NIL)
- Why are they using these methods to correct their vision? (free text)
- Did any eye care specialist recommend any Myopia Control options? (Yes, No, NA)
- May I know what have they recommended? (free text)
- What influenced you in choosing the types of vision correction for your child? (multiple options could be selected)
- recommended by friends/family
- recommended by social media
- recommended by your eye care specialists
- advertisements
- due to superstition/traditional reasons
- affordability in the long run
- family consent
- What do you think will work best for Myopia Control? (Ranking: Normal spectacles, orthokeratology lenses, myopia control lenses, atropine, multifocal soft contact lenses, normal soft contact lenses, RGP [hard lens], outdoor activities, control the amount of time doing near work, nutrition, Ayurveda, Tradition Chinese Medicine).

Participation was voluntary and anonymous in the survey. The explanation for the research was explained in the message that was sent out and before the beginning of the survey. The data was collected between April 2020 and May 2020.

### Statistical Analysis

Statistical analysis was conducted with IBM SPSS Statistics for Windows, Version 27.0 (IBM Corp, New York, USA). Count and proportion were calculated for each question response, and comparison was done using the chi-square test with *p* < 0.05.

## Results

### Responses

A total of 130 complete survey responses were received from the professional groups. Of the study participants, 32 (24.6%) were ophthalmologists, 91 (70.0%) were optometrists, 2 (1.5%) were opticians and 5 (3.8%) were optometrist students. For the survey on parents' opinion, a total of 138 parents responded to the survey, of which 99 (71.7%) of them had at least one myopic child.

### Frequency of Prescribing Myopia Correction

**Figure 1:**
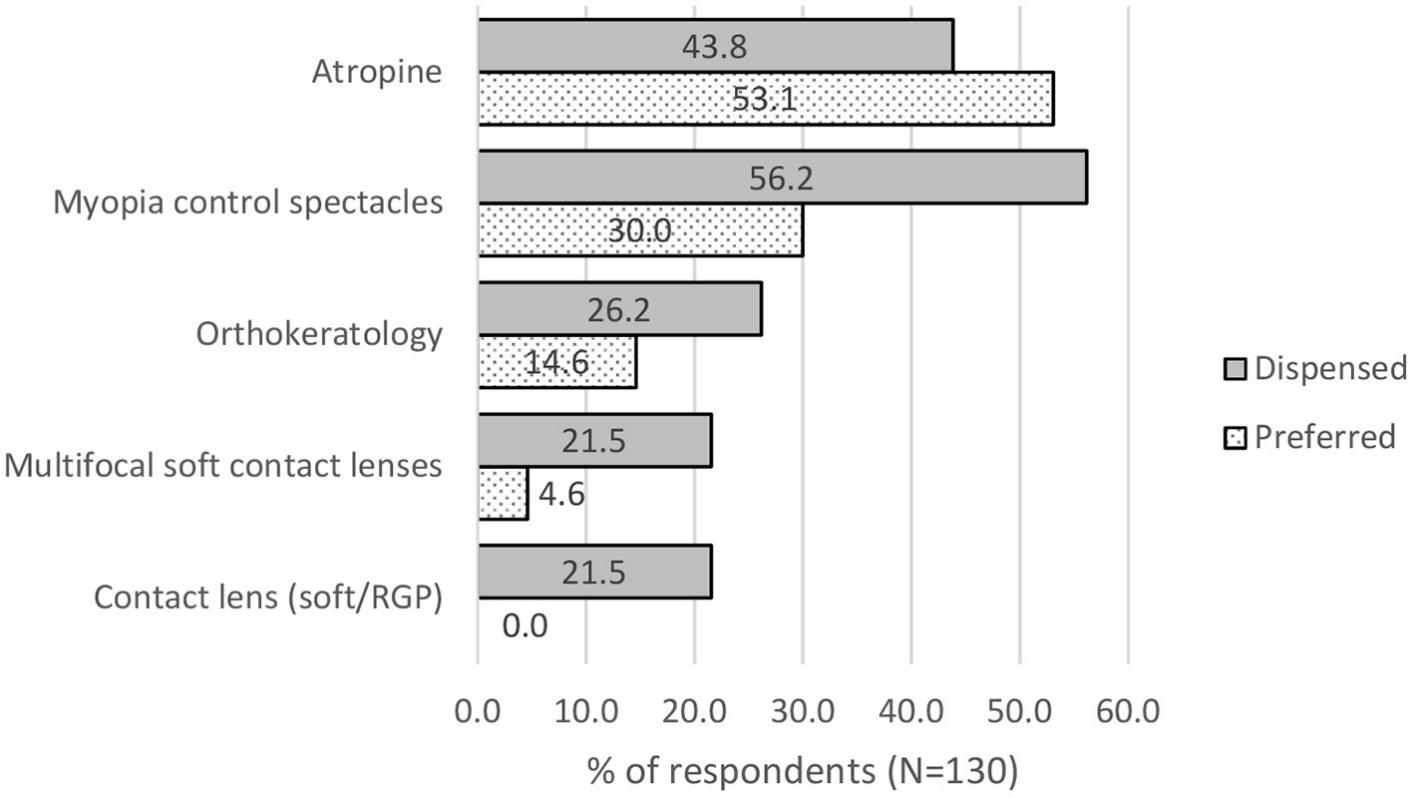
Preference and actual dispensing of the type of myopia control products by eye care practitioners.

Majority of the practitioners (80.0%), do prescribe myopia control intervention to myopic patients; *X*^2^(0) = 416, *p* < 0.001. However, only 33.1% did so during the first visit; *X*^2^(0) = 21, *p* < 0.001). Overall, most practitioners preferred myopia control spectacle lenses (30.0%) and atropine (53.1%) as myopia control interventions; *X*^2^(3) = 3,477, *p* < 0.001. As such, most of them dispensed myopia control spectacle lenses (56.2%), followed atropine (43.8%) and orthokeratology (26.2%) in real life (see Figure 1).

**Figure 2:**
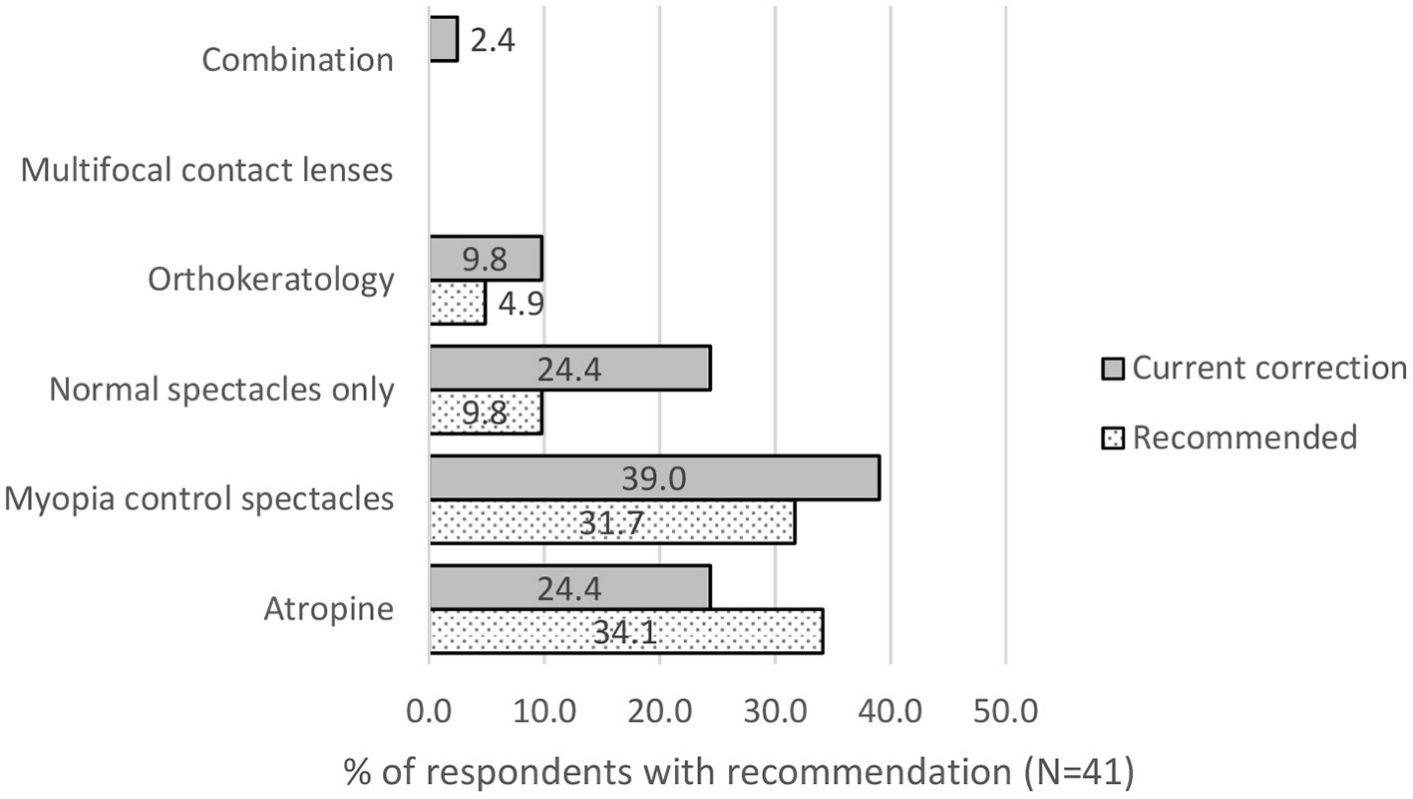
Out of those parents recommended to use myopia control, which product was most recommended (grey bars) by eye care professionals and used (dotted bars) by respondents.

According to the survey on 99 parents with myopic children, 41.4% were recommended to use myopia control interventions by their eye care practitioners; *X*^2^(1) = 82, *p* < 0.001. Of which, 24.4% were recommended to use atropine, 39.0% of them were advised to use myopia control spectacle lenses and 9.8% were recommended orthokeratology. The rest were recommended other interventions like increasing outdoor time, reducing near work, looking at green pasture and blue-cut lenses, Figure 2, gray bars. Out of those that were recommended to use myopia control interventions, 75.6% were using myopia control interventions. Of which, 39.0% used myopia control spectacle lenses, 9.8% used orthokeratology, 24.4% used atropine and 2.4% used combination treatment (see Figure 2), gray bars. Despite the recommendation from eye care practitioners, even up to 24.4% of those given myopia control recommendations used single vision lenses (spectacle or soft contact lenses) for correction.

### Factors Influencing Parents' Decision to use Myopia Control Options

**Figure 3:**
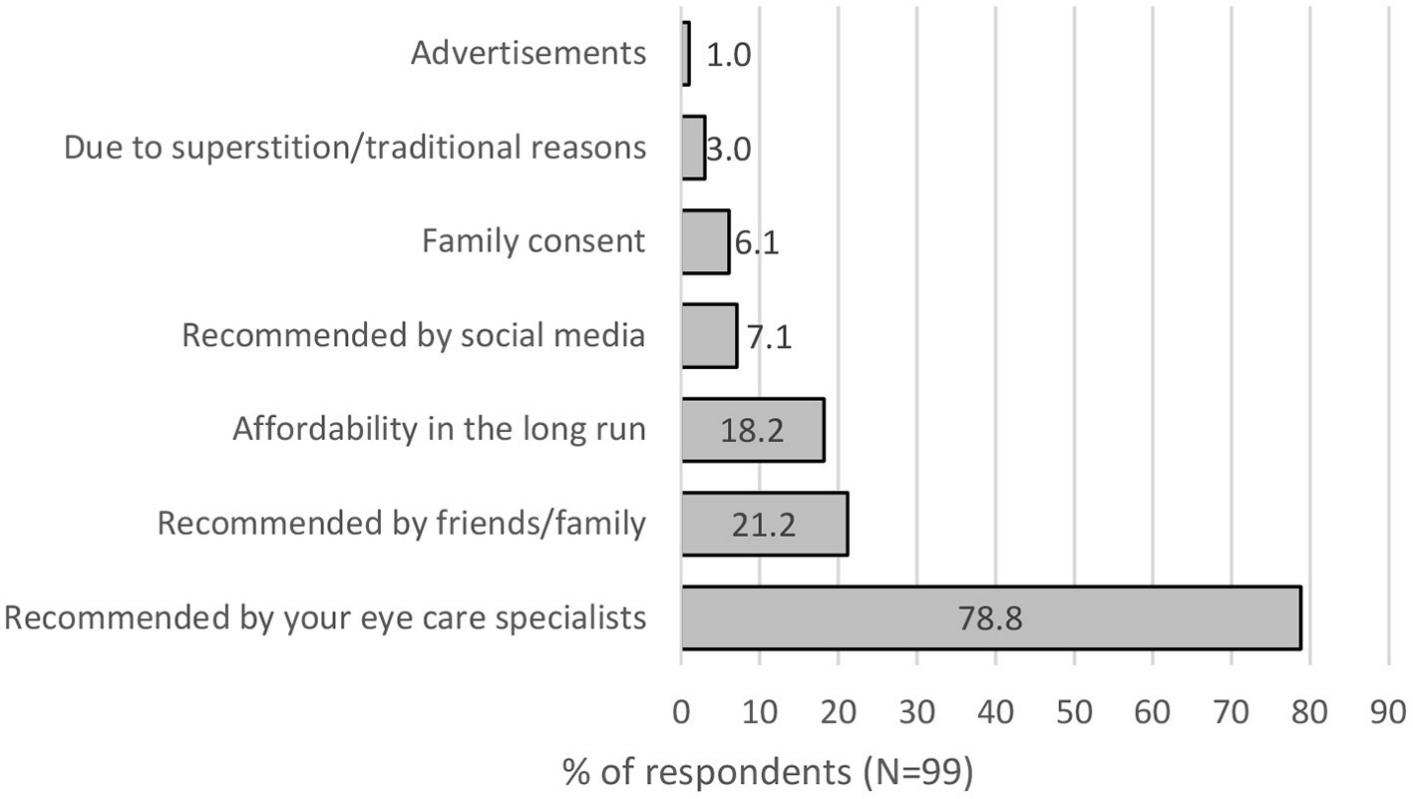
The factors that will influence parents to use the type of vision correction.

78.8% of the parents would follow what the eye care practitioners recommended, with only 21.2% taking advice from their friends or family members. 18.2% needed to have affordability in the long run, and 7.1% would follow social media or get consent from their family (see Figure 3).

### Perceived Effectiveness of Myopia Control Options by Parents

**Figure 4:**
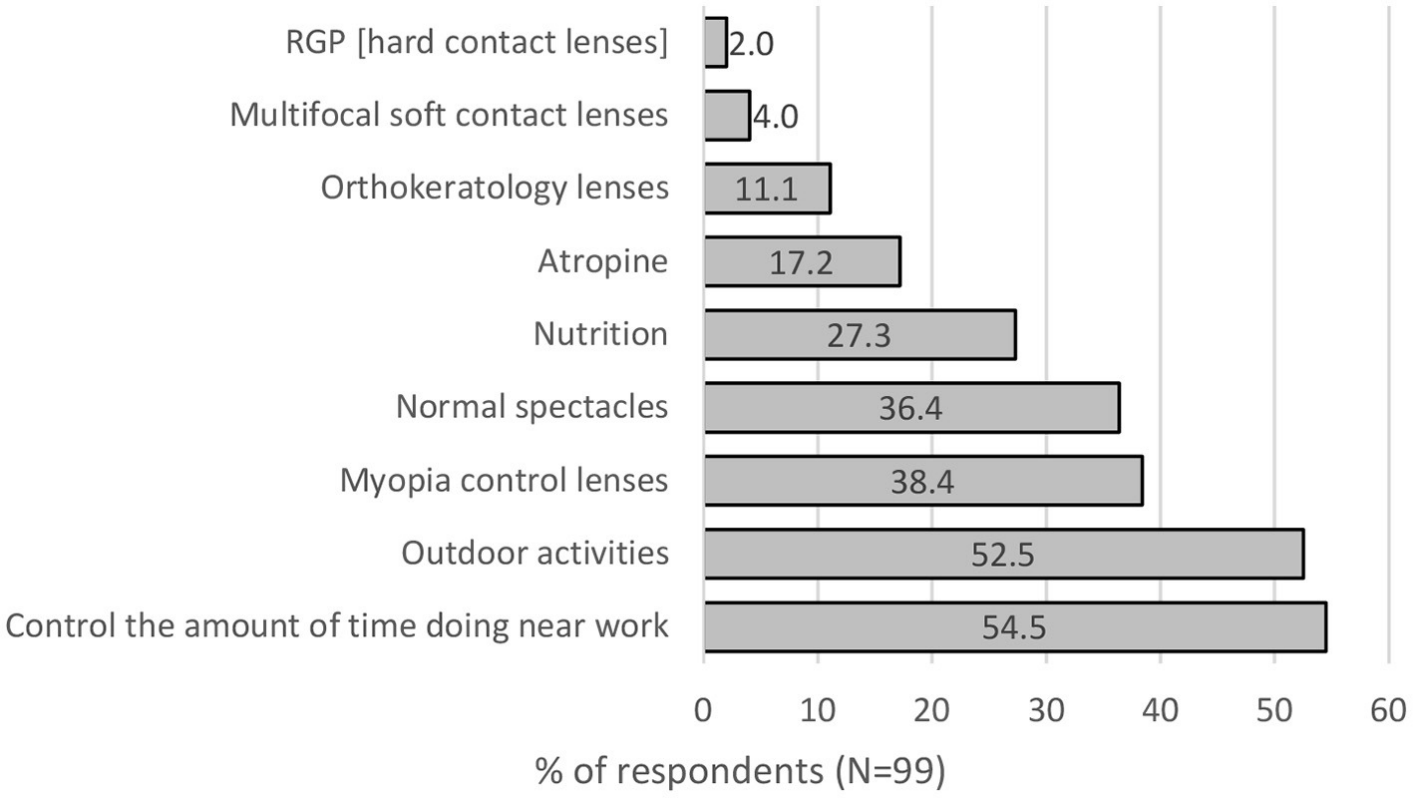
Perceived effectiveness of myopia control interventions by parents.

Parents perceived increasing outdoor time (52.5%) and controlling the amount of near work (54.5%) to be effective myopia control options. 38.4% perceived myopia control spectacle lenses and single vision lenses (36.4%) to be the best for myopia control. Followed by nutrition (27.3%), Atropine (17.2%) and orthokeratology (11.1%) (see Figure 4).

### Factors Preventing the Prescription of a Myopia Control Intervention

**Figure 5:**
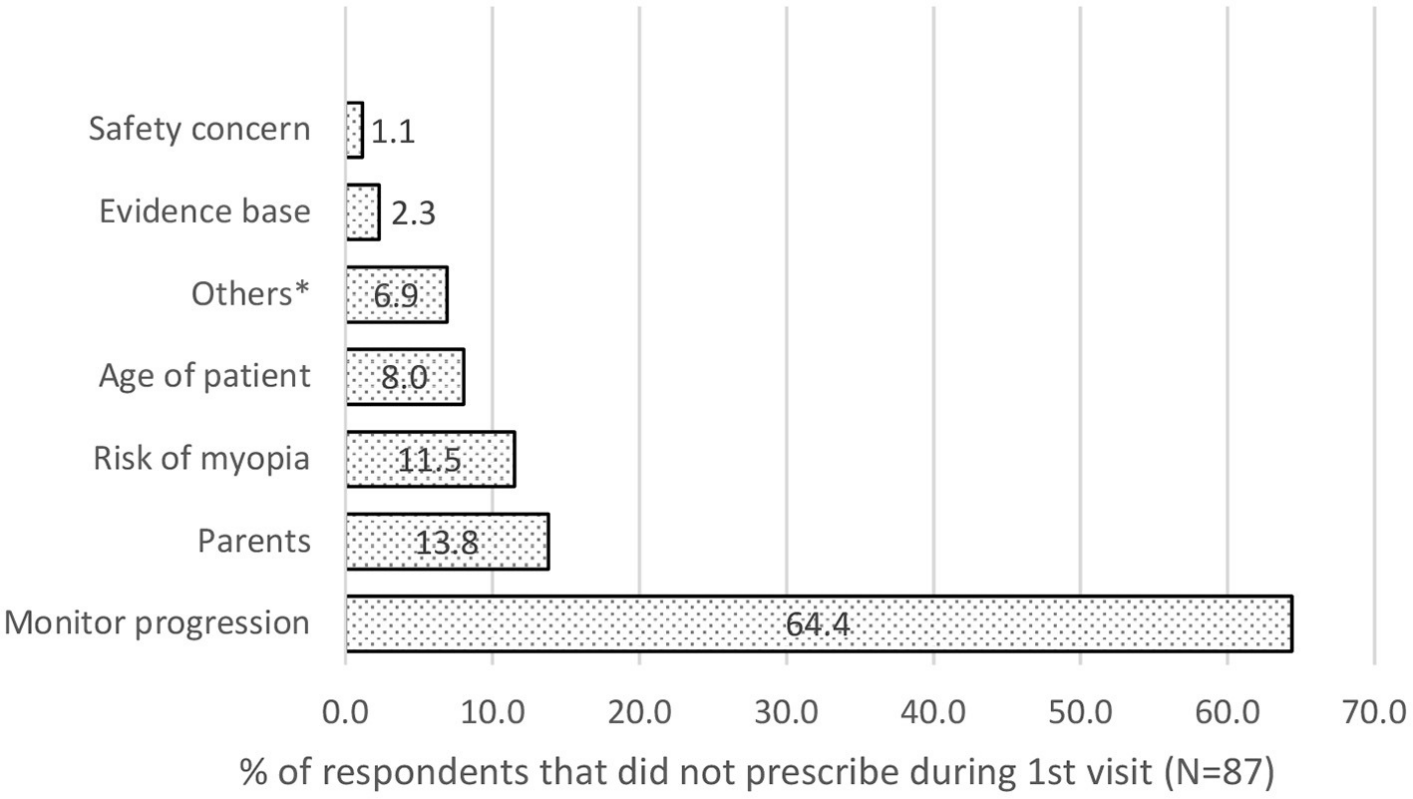
Main factors preventing Eye care practitioners from prescribing myopia control intervention during the first visit.

64.4% wanted to monitor the rate of myopia progression first before prescribing myopia control intervention. 13.8% were worried that parents cannot afford it, 8.0% wanted to look at the age and 11.5% wanted to assess the risk of myopia first. Other 2.3% needed more evidence that the product would work. In comparison, the remaining 6.9% would advise behavioral change like less near work and more outdoor time, ensure good binocular vision and find it easier to give single vision lenses and 1.1% were concerned about the safety of the product (see Figure 5).

### Factors That Will Help Eye Care Practitioners to Prescribe Myopia Control Interventions

**Figure 6:**
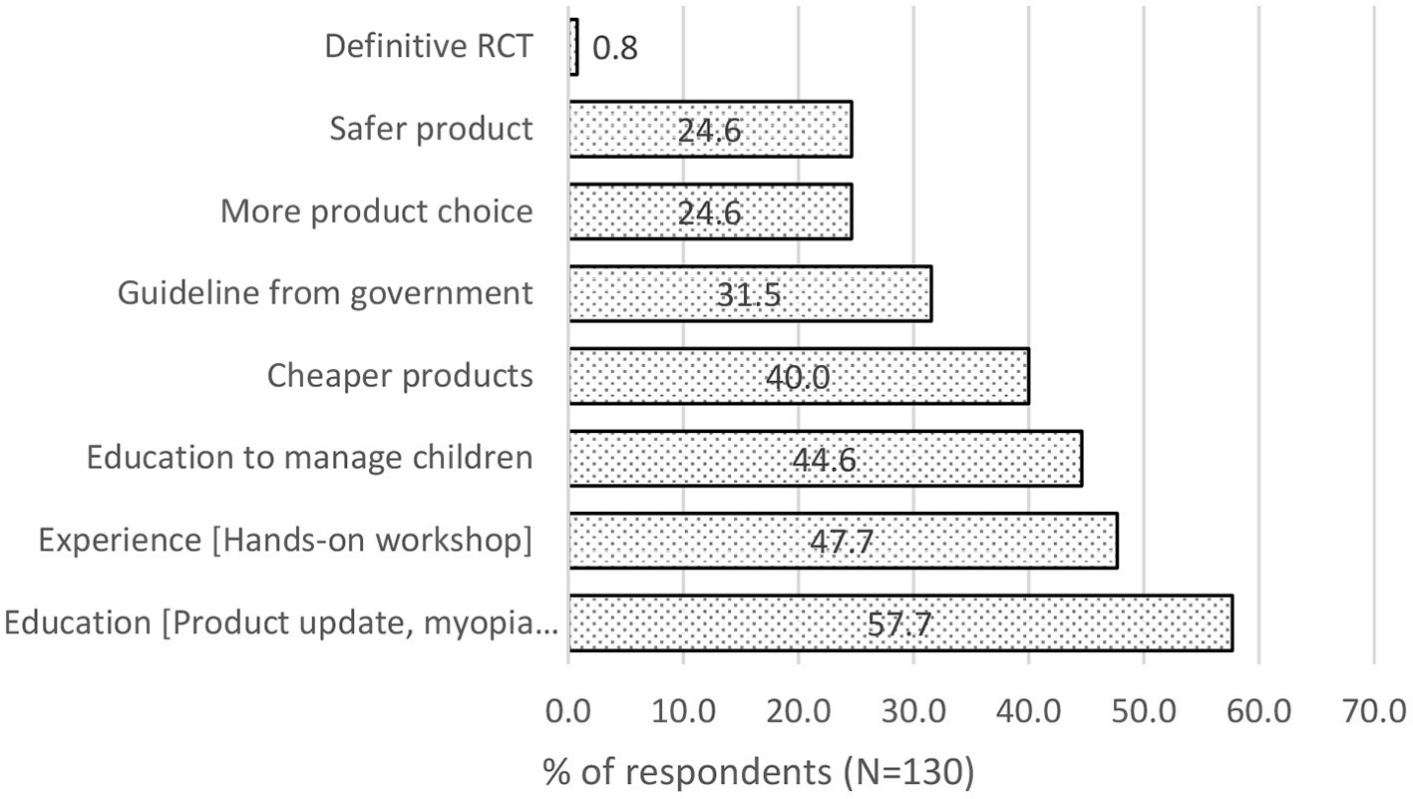
Factors that will encourage Eye care practitioners to prescribe myopia control interventions.

Most (57.7%) felt that having more education on myopia management and product would encourage them to prescribe myopia control interventions. 47.7% also felt that a hands-on workshop to experience the fitting of myopia control interventions would give them more confidence. 44.6% also wanted more education on managing children as they lack experience in pediatric optometry/ophthalmology (see Figure 6).

## Discussion

This study examines the self-reported attitudes and practices of eye care practitioners and parents' opinions toward myopia control in Singapore. One hundred and thirty eye care practitioners responded out of about 2,000 registered opticians and optometrists and about 300 registered ophthalmologists in Singapore. That is about 7.4% of registered optometrists and 10.7% of registered ophthalmologists in Singapore. The exact response rate was unknown as we could not measure the number of eye care practitioners who had received the questionnaire. It may be presumed that questionnaires are completed by people interested in myopia control of myopia in general based on 80.0% of them prescribing myopia control intervention in their practice. The type of myopia control prescribed by each eye care practitioner was in line with the healthcare regulation in Singapore. The ophthalmologists would mainly prescribe atropine as it can only be done by ophthalmologists under the Singapore Medicine Act and Optician and Optometrist act (20, 21). Nevertheless, the regulation did not stop them from recommending interventions that they cannot prescribe as they could refer out to practitioners that prescribe them.

A study on pediatric ophthalmologists in Germany showed that 57% routinely treat to slow down myopia progression and 74.8% do so if the progression rate was 1 dioptre or more per year. The most common treatment used were atropine 0.01% (63.4%), followed by atropine 1% (10.9% and atropine 0.5% (8.9%). In addition, most of them also recommend more outdoor time (86%) and less screen time (60.2%) (22). This is similar to the outcome of this survey where 40.63% of ophthalmologists treated myopia at the first visit with 62.5% of them monitoring the rate of progression first. Though 100% of them prescribes atropine, 96.9% preferred atropine as the first choice, 78.13% preferred myopia control spectacles as the second choice and 56.3% selected multifocal contact lenses as the third choice.

Out of 138 parents, 99 have at least one myopic children responded to the questionnaire for their opinions about myopia control. Similar to the questionnaire for eye care practitioners, the exact response rate could not be adequately estimated. It was presumed that parents who responded to the questionnaire are interested in myopia and are assumed to be more aware of myopia control methods.

Though 80.0% of the eye care practitioners prescribe myopia control interventions, only 33.1% do so for patients who visited them for the first time. This outcome was also reflected in the parents' questionnaire, with only 41.4% given any recommendation. The main reason (56.6%) for not dispensing myopia control intervention during the first visit as 64.4% of them wanted to monitor the rate of myopia progression. However, there was a lack of myopia progression data from a first-time patient. This is especially true for first-time wearers of visual correction. If parents do not return to the same practice, they will always be first-time patients in another practice.

Atropine was the preferred option in the opinion of 53.1% of eye care practitioners but 52.5% of parents preferred more outdoor time. From this study, if eye care practitioners gave parents advice, most of them (75.6%) took the advice and used myopia control interventions like myopia control spectacles, atropine and orthokeratology. However, all parents who were not given any advice had normal spectacle lenses or soft contact lenses (single vision lenses) prescribed for their children. Without any prior knowledge and proper advice from eye care practitioners, normal spectacle was the next preferred choice of most parents. Parents' responses to factors that influence their choice of vision correction further proved that eye care practitioners' recommendation is pivotal in parents' decision to use myopia control intervention for their children as 78.8% of parents were influenced by eye care practitioners' recommendation.

13.8% of eye care practitioners were worried that parents could not afford myopia control intervention, contrary to the findings that 78.8% of parents were influenced by eye care practitioners' recommendations and only 18.2% of parents were concerned about affordability in the long run. Concern about cost by eye care practitioners was similar to the global survey conducted by Wolffsohn et al. (16, 17), where the main reason for not prescribing myopia control intervention was due to cost (20.6%), followed by inadequate information about modalities (17.6%) and unpredictable outcomes (9.6%).

Finally, most eye care practitioners felt that continuous education on myopia management products and hands-on workshops would give them more confidence and experience to prescribe them in their practice. Ability to manage young patients was also crucial since myopia onset usually happens at a young age (23). Despite the vast volume of research evidence for myopia management (14), a lack of confidence in appraising studies (24) and insufficient time in clinical practice (25) are possible reasons for eye care practitioners to not read up scientific publications on myopia control. Moreover, eye care practitioners have the added benefit of accruing points from education programs to maintain their professional license instead of learning by themselves through reading scientific publications on myopia control. Similar to Australia, there is an absence of clinical guidelines for myopia control from regulatory bodies like the Optometrists and Opticians Board in Singapore. However, the lack of regulatory approved guidelines was not the main concern (31.5%) in Singapore, unlike the study in Australia by Douglass et al. (18). Though there are published guidelines that recommend that myopia onset at a younger age should be offered myopia control intervention (15), and the type of intervention could be based on the rate of myopia progression (14). There was no clear definition in the published guidelines on minimum age, degree of myopia and rate of myopia progression for eye care practitioners to recommend myopia control interventions and criteria for ceasing treatments. This is evident in the findings of different regions in response to the minimum age and amount of myopia to prescribe myopia control (17, 18, 20, 21). Hence, further studies are needed to have an improved guideline to provide more consistent and evidence-based care.

As this study was conducted within a short period, the sample size was small and may not be representative of the whole eye care community in Singapore. Due to the options designed in the questionnaire to focus more on myopia control interventions, outdoor time, screen time and near work was not included in the multiple choices. As such this study was not able to investigate the awareness of outdoor time, screen time and near work for myopia control in parents and eye care practitioners. Nevertheless, there was a previous study done to establish that 87.7% of parents in Singapore were aware of the protective role of outdoor activity in myopia development and progression (26). This was mostly attributed to the public education done by the National Myopia Prevention Program together with other representatives from the Ministry of Education, Singapore Armed Forces, Ministry of Social and Family Development, National University of Singapore, Singapore Eye Research Institute, Optometrists and Opticians Board, Ministry of Health, and Singapore optometric and professional groups (27). Whether awareness translates to actual practice will be interesting to investigate further.

## Conclusions

This study where questionnaires were given to eye care practitioners and parents in Singapore identified that eye care practitioners are the critical influence for prescribing myopia control. Uptake of myopia control can improve if more education is given to eye care practitioners for more updates and experience with the products for and management of myopic children. The cost of myopia control is of concern to eye care practitioners. Hence health economic evaluation should be done to understand the cost-effectiveness of using myopia control interventions to prevent myopia related pathology. Despite the availability of published guidelines on myopia management, further research is required to establish the minimum age, amount of myopia, and progression to start prescribing myopia control intervention, age to cease treatment or give myopia control to all myopic patients at the first visit. Regulatory approval of myopia guidelines should be considered to support the change in behaviour in eye care practitioners on myopia management.

## Data Availability Statement

The raw data supporting the conclusions of this article will be made available by the authors, without undue reservation.

## Ethics Statement

Ethical review and approval was not required for the study on human participants in accordance with the local legislation and institutional requirements. Written informed consent for participation was not required for this study in accordance with the national legislation and the institutional requirements.

## Author Contributions

AY: conceptualization, methodology, supervision, project administration, and writing—original draft preparation. AY and BD: validation. AY and BP: formal analysis. AY and PV: investigation. BD: resources. BD and BP: writing—review and editing. All authors have read and agreed to the published version of the manuscript.

## Conflict of Interest

AY, BP, and BD are employees of Essilor International. The remaining author declares that the research was conducted in the absence of any commercial or financial relationships that could be construed as a potential conflict of interest. The authors declare that this study received funding from Essilor International. The funder was involved in the study design, collection, analysis, interpretation of data, the writing of this article and the decision to submit it for publication.

## Publisher's Note

All claims expressed in this article are solely those of the authors and do not necessarily represent those of their affiliated organizations, or those of the publisher, the editors and the reviewers. Any product that may be evaluated in this article, or claim that may be made by its manufacturer, is not guaranteed or endorsed by the publisher.
